# Broken File Retrieval in the Lower Right First Molar Using an Ultrasonic Instrument and Endodontic Micro Forceps

**DOI:** 10.1155/2019/7940126

**Published:** 2019-10-31

**Authors:** Ratna Meidyawati, Endang Suprastiwi, Hasti Dwi Setiati

**Affiliations:** Department of Conservative Dentistry, Faculty of Dentistry, Universitas Indonesia, Indonesia

## Abstract

Broken files affect cleaning, shaping, and filling processes of the root canal, thereby causing maintenance failure. *Objective*. This report explains how to remove broken files using ultrasonic instruments and endodontic micro forceps. *Case Report*. A 25-year-old female patient had incomplete root canal treatment at the lower right first molar 1 week ago. There were radiolucency in the bifurcation and apical root and the presence of broken files in the 1/3 coronal mesiolingual root. The retrieval started by making a staging platform with an ET20 ultrasonic tip. Endodontic micro forceps were used including a screw wedge that works by clamping the file fragments through a mechanical lock and pulling them to the coronal. *Conclusion*. It is possible to successfully remove broken files from the root canal using ultrasonic instruments and endodontic micro forceps.

## 1. Introduction

The success of root canal treatment depends on the results of the cleaning and shaping process. However, there is a risk of broken files because its presence inhibits the process of cleaning, shaping, and filling, thereby leading to treatment failure [1]. A broken file often occurs in the molar teeth, especially at the lower jaw because of poor access, small diameter, and sharp curvature of the root canal. Both hand instruments and machine root canal instruments are mostly made of stainless steel and nickel titanium; therefore, there is a potential that they might break. It has been recorded that the incidence of broken files is 0.25% for hand instruments and 1.68%-2.4% for rotary instruments [2].

There are several alternative treatments for this occurrence, and they include taking the broken file fragment and bypassing it while inside the root canal [1]. However, there are several factors considered in managing these cases, and they include visibility, the location of broken file teeth, and the structure of the remaining tooth tissue [1]. Furthermore, this treatment often requires special assistance because of the risk of complications such as pushing the file apically, extruding fragments outside the apex, risk of tooth fracture due to dentin uptake excess, root perforation, and the occurrence of a ledge [3].

Technological advancement has made it possible to have several tools for file retrieval, including ultrasonic, microtube, and plier devices, with the assistance of a microscope to facilitate visibility and minimize the extraction of root canal dentine [1]. Therefore, this report discussed the management of broken file cases in the mandibular molars by using an ultrasonic device and endodontic micro forceps with the assistance of a microscope.

## 2. Case Report

**Figure 1:**
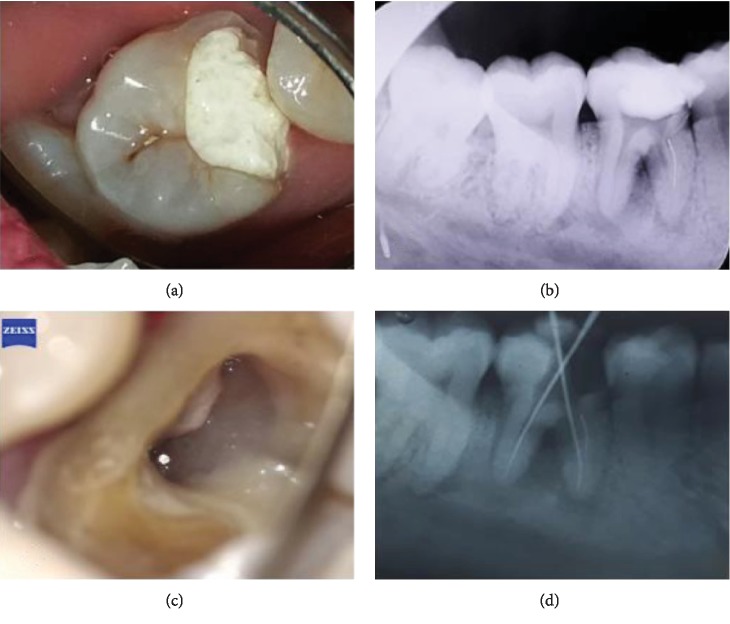
(a) Preoperative clinical feature. (b) Preoperative radiograph with a broken file in the mesiolingual canal. (c) Broken file was seen inside the canal (dental microscope magnification). (d) Ensuring the position of the file with an instrument.

A 25-year-old female patient complained that her lower right back teeth are not comfortable with chewing; therefore, root canal treatment was conducted one week ago after one year of spontaneous pain. On intraoral examination, caries reached the pulp in the lower right first molars, with negative vitality and positive percussion. Radiographic examination showed radiolucent images in the bifurcation area and apical mesial and distal roots and a broken file from the orifice to the middle of the mesiolingual root (Figures 1(a) and 1(b)).

Based on the subjective, objective, and radiographic examination, the diagnosis of the right mandibular first molar is symptomatic apical periodontitis, accompanied by a broken file on the mesiolingual root. The treatment conducted was for the nonvital root canals with Ceramage (Shofu Inc., Japan) onlay restoration.

The tooth was prepared to obtain adequate coronal access with 2.5% NaOCl as an irrigant to remove debris. The working length was measured through the use of an electronic apex locator (Root ZX II, Morita). The root canal was prepared in the mesiobuccal and distal roots through the use of ProTaper Next (Dentsply Maillefer, Switzerland) until the master apical file was obtained at X3/16.5 mm mesiobuccal root and X3/17.5 mm distal root. Irrigation was conducted, and the orifices of both root canals were closed with a paper point and cotton to prevent the entry of file fragments. In the mesial root canal, the retrieval started by making a staging platform with a Satelec ET20 (Satelec Acteon, France) ultrasonic tip until 2 to 3 mm of the broken file was exposed. This was aimed at loosening the file from the root canal wall of the dentin and providing a space for the device. The staging platform is the space between the tip of the exposed file and the root canal wall which was further circulated around the file in an anticlockwise direction to give the effect of unscrewing force. This helps in retrieving files with a clockwise cutting action. The energy applied can help loosen the file and provide a space between it and the root canal wall. The Satelec ET25 (Satelec Acteon, France) ultrasonic tip could then be used to loosen those on the part of the wall.

**Figure 2:**
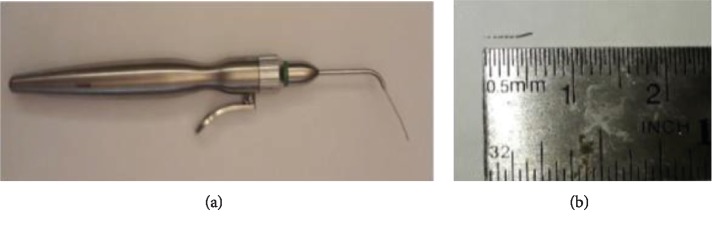
(a) Endodontic micro forceps. (b) Broken file was successfully retrieved.

Irrigation was conducted by using 2.5% NaOCl and 17% EDTA, and activation was done through an EndoActivator (Dentsply Maillefer, Switzerland). Since the direct application of ultrasonic devices does not have the ability required to remove the file, endodontic micro forceps (Broken Instrument Removal Kit, Zumax, China) were used including a screw wedge that works by clamping the file fragments through a mechanical lock and pulling them to the coronal (Figure 2(a)). After the file fragment has been successfully lifted (Figure 2(b)), it was confirmed with a photo of the radiograph.

The mesiolingual root canal was prepared through the ProTaper Next (Dentsply Maillefer, Switzerland) up to X3/16.5 mm and the master gutta-percha cone confirmed by radiographic photographs.

It was medicated with calcium hydroxide paste (Calcipex®, Nippon Shika-Yakuhin, Shimonoseki, Japan) and restored temporarily.

Two weeks after the first visit, the root canal was filled with continuous wave compaction through gutta-percha (ProTaper Next*®* Gutta Percha, Dentsply Tulsa Dental, Switzerland) with an MTA Fillapex sealer and closed with RMGIC (Fuji II LC, GC, Japan) and temporary restoration (Caviton, GC Corporation, Japan).

**Figure 3:**
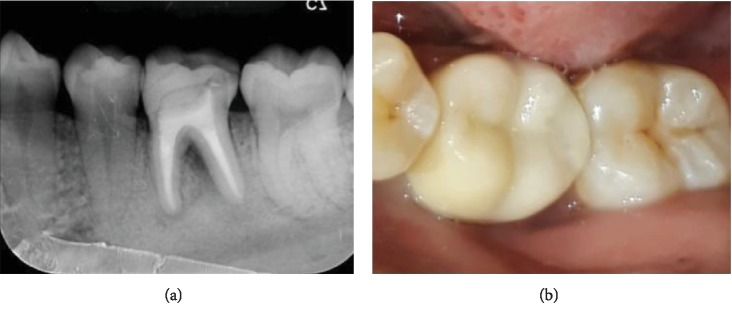
One-month postoperative evaluation: (a) postoperative radiograph; (b) Ceramage® onlay for restoration.

One week after obturation, the preparations were completed to make Ceramage® onlay (Shofu Inc., Japan). Two weeks after onlay preparation, onlay was cemented using resin cement (Figure 3(b)). The evaluation after one month showed negative on subjective examination and the percussion and palpation test. The radiograph showed bifurcation, and apical radiolucency was reduced.

## 3. Discussion

The use of an ultrasonic instrument assisted by a microscope is a conservative method of handling a broken file compared to other alternatives [4, 5]. It can erode the structure of the dentine conservatively and is less likely to damage the root structure and periodontal tissue [5]. However, its application was unable to loosen the file until it reached the coronal; therefore, a tool was needed to clamp the file and draw its fragments in the coronal direction through a microtube with either a screw wedge or a loop device (DentalCadre, Seattle, WA) [2]. This is common in broken files longer than 4.5 mm or visible under ultrasonic activation but could not be retrieved by the device [2].

Removal of broken files can be conducted in dry or wet conditions [2]. Dry conditions provide better visibility with a microscope, thus preventing procedural errors [2]. However, heat generated from ultrasonic vibrations is unavoidable, and the temperature has the possibility to increase to more than 10°C on the external root surface causing damage to the periodontal tissue [2]. The files are also susceptible to secondary heat if the ultrasonic tip is in contact with the file [2]. Therefore, EDTA irrigation was conducted when the ultrasonic tip was activated at the lowest power setting [2]. This improved the cleanliness of the root canal wall [2].

The tips used were ET20 and ET25 from the Endo Success*™* Retreatment Kit (Satelec Acteon, France), made from titanium niobium alloy and coated with diamond, making them abrasive. The ET20 was moved counterclockwise in 1/3 coronal and ET25 in the middle 1/3 of the root canal to give the file an unscrewing force effect [2].

The root canal was filled with a continuous wave compaction technique and through the use of an MTA Fillapex (Angelus, Brazil) sealer which is good for dentin because of its ability to harden. They also possess good density, thus preventing leakage in the periapical region and closing communication between pulp and periodontal tissue. The MTA content in the sealer supports healing and hard tissue formation in the lesion.

MTA Fillapex has several advantages such as the stimulation of new tissue formation, rapid tissue repair without causing inflammatory reactions, high radio-opacity, provision of good visualization on the radiograph, release of calcium ions to induce rapid tissue regeneration in areas with periapical lesions and microbial activity, making insertion and handling easier, having adequate working time, and being easy to remove (on retreatment, especially if used with gutta-percha) [6, 7]. It can also be used for antimicrobial activity against *M. luteus*, *S. aureus*, *E. coli*, *P. aeruginosa*, *C. albicans*, and *E. faecalis* because of its alkaline pH [7, 8].

In this case, the mandibular right first molars with symptomatic apical periodontitis with a broken file on the orifice to the middle of the mesiolingual root canal were retrieved with a combination of ultrasonic tips and endodontic micro forceps, accompanied by a microscope to increase visibility. The treatment was successful because a broken instrument was completely removed followed by loss of subjective complaints. The radiograph also revealed diminished apical radiolucency.
